# “FLipping” the Story: FLT3-Mutated Acute Myeloid Leukemia and the Evolving Role of FLT3 Inhibitors

**DOI:** 10.3390/cancers14143398

**Published:** 2022-07-13

**Authors:** Tristan E. Knight, Holly Edwards, Soheil Meshinchi, Jeffrey W. Taub, Yubin Ge

**Affiliations:** 1 Cancer and Blood Disorders Center, Seattle Children’s Hospital, Seattle, WA 98105, USA; smeshinc@fredhutch.org; 2 Division of Hematology and Oncology, Department of Pediatrics, University of Washington School of Medicine, Seattle, WA 98105, USA; 3 Department of Oncology, Wayne State University School of Medicine, Detroit, MI 48202, USA; pitmanh@karmanos.org (H.E.); gey@karmanos.org (Y.G.); 4 Molecular Therapeutics Program, Barbara Ann Karmanos Cancer Institute, Wayne State University School of Medicine, Detroit, MI 48201, USA; 5 Fred Hutchinson Cancer Research Center, Seattle, WA 98109, USA; 6 Division of Hematology/Oncology, Children’s Hospital of Michigan, Detroit, MI 48201, USA; jtaub@med.wayne.edu; 7 Department of Pediatrics, Wayne State University School of Medicine, Detroit, MI 48202, USA; 8 Department of Pediatrics, Central Michigan University, Mt. Pleasant, MI 48859, USA

**Keywords:** acute myeloid leukemia, AML, crenolanib, FLT3, FLT3 inhibitor, gilteritinib, midostaurin, quizartinib, sorafenib

## Abstract

**Simple Summary:**

Patients with acute myeloid leukemia (AML) may have a number of different mutations. Those with mutations in the *FLT3* gene have a higher risk of relapse and death than those lacking these mutations. FLT3 is a key receptor on the surface of AML cells, which drives cell survival and growth. Although activation of this receptor is normally tightly controlled, in AML, *FLT3* mutations allow it to activate itself, independent of external control. Over the past 5 years, a number of new drugs have been developed to specifically target these mutations. In this article, we discuss these drugs and their uses, as well as the mechanisms by which AML cells may gain resistance to them and how that resistance can be overcome.

**Abstract:**

The treatment of many types of cancers, including acute myeloid leukemia (AML), has been revolutionized by the development of therapeutics targeted at crucial molecular drivers of oncogenesis. In contrast to broad, relatively indiscriminate conventional chemotherapy, these targeted agents precisely disrupt key pathways within cancer cells. *FMS-like tyrosine kinase 3* (*FLT3*)—encoding a critical regulator of hematopoiesis—is the most frequently mutated gene in patients with AML, and these mutations herald reduced survival and increased relapse in these patients. Approximately 30% of newly diagnosed AML carries an *FLT3* mutation; of these, approximately three-quarters are internal tandem duplication (ITD) mutations, and the remainder are tyrosine kinase domain (TKD) mutations. In contrast to its usual, tightly controlled expression, FLT3-ITD mutants allow constitutive, “run-away” activation of a large number of key downstream pathways which promote cellular proliferation and survival. Targeted inhibition of FLT3 is, therefore, a promising therapeutic avenue. In April 2017, midostaurin became both the first FLT3 inhibitor and the first targeted therapy of any kind in AML to be approved by the US FDA. The use of FLT3 inhibitors has continued to grow as clinical trials continue to demonstrate the efficacy of this class of agents, with an expanding number available for use as both experimental standard-of-care usage. This review examines the biology of FLT3 and its downstream pathways, the mechanism of FLT3 inhibition, the development of the FLT3 inhibitors as a class and uses of the agents currently available clinically, and the mechanisms by which resistance to FLT3 inhibition may both develop and be overcome.

## 1. Introduction

Acute myeloid leukemia (AML) is a malignancy characterized by the aggressive, malignant clonal expansion of immature myeloid-lineage cells [1]. It accounts for approximately one-third of all leukemia diagnoses among adults and nearly half of leukemia-related deaths [2]. It is somewhat less common in children, making up approximately one-fifth of all acute leukemia diagnoses but accounting for over half of leukemia-related deaths [2]. Despite ongoing advances in therapy and steadily improving outcomes, just over a quarter of adults diagnosed with AML will be alive 5 years after diagnosis; in children, approximately one-third will have died of their disease within 5 years of diagnosis [2]. However, the prognosis and clinical outcomes of this disease vary widely among patients, and mortality is not uniform across disease subtypes. Part of this variance is due to patient-related factors such as age and clinical performance status [3]. Genetic features, including both molecular and cytogenetic (e.g., chromosomal) abnormalities, are also critically important, to the extent that modern AML classification schemata are predicated on the identification of these mutations [4]. 

*FMS-like tyrosine kinase 3* (*FLT3*) gene mutations are the most common cytogenetic abnormality seen in patients with AML and are present in up to one-third of newly diagnosed patients [5]. Two primary classes of *FLT3* mutations exist. Internal tandem duplication (FLT3-ITD) within the juxtamembrane domain is the most common (approximately 25% of all patients), followed by the tyrosine kinase domain (FLT3-TKD) (approximately 6–8% of all patients) [5]. Patients with *FLT3*-TKD mutations appear to have a broadly similar prognosis to those with *FLT3* wild-type (FLT3-WT) [6,7,8]. Patients with *FLT3*-ITD mutations, however, have a notably poorer prognosis, and the presence of these mutations heralds a reduced survival rate and increased risk of relapse [9,10,11]. FLT3 is, therefore, an inviting target for pharmacologic inhibition or disruption, and in recent years, a number of promising agents have been developed for precisely this purpose. The first such agent, midostaurin, was approved by the United States’ Food and Drug Administration (FDA) in April 2017 for use in newly diagnosed patients with *FLT3*-mutated AML [12]. 

As existing FLT3 inhibitors gain increasing use, and as new FLT3 inhibitors continue to be developed, resistance to this class of agents is becoming more widespread and poses a significant challenge. This review, therefore, examines the biology of FLT3 and its downstream pathways, explores the mechanism of FLT3 inhibition and development and implementation of FLT3 inhibitors, and assesses the mechanisms by which resistance to FLT3 inhibition may both develop and be overcome.

## 2. The FLT3 Receptor

**Figure 1:**
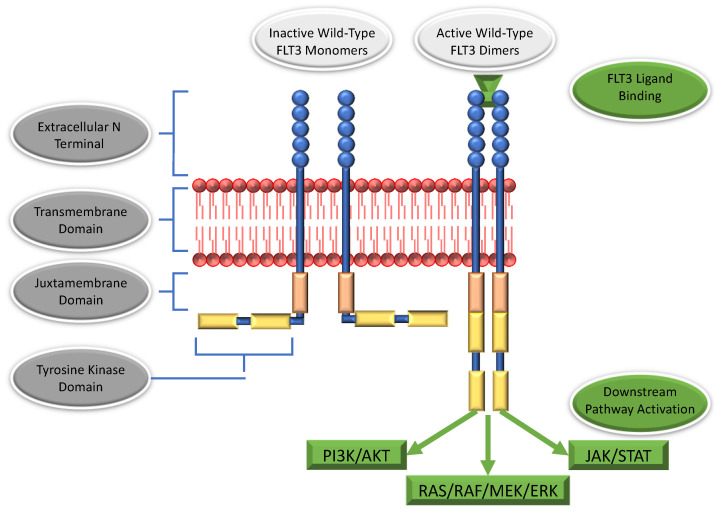
Wild-type FLT3 is held in an inactive, monomeric conformation. Upon FLT3 ligand binding, FLT3 dimerizes, auto-phosphorylates, and undergoes a conformational change, leading to activation of downstream signaling pathways associated with cellular growth, proliferation, and survival.

FLT3 is encoded by the *FLT3* gene, which is located at 13q12 and contains 24 exons extending over a region of at least 100 kilobases [13,14]. The resultant protein is membrane-bound and comprised of 993 amino acids with a combined total molecular weight of approximately 160 kDA [13]. FLT3 is a ligand-activated, class-3 tyrosine kinase receptor (e.g., a member of the platelet-derived growth factor receptor (PDGFR) subfamily) with close structural homology to PDGFR alpha and beta, c-Kit, and colony-stimulating factor 1 receptor (CSF1R) [14,15]. Upon binding to its ligand (FLT3 ligand; FL), FLT3 undergoes homodimerization and conformational changes—specifically, the intracytoplasmic kinase tails unfold and undergo autophosphorylation, resulting in FLT3 activation (See Figure 1) [16]. Following ligand binding, the dimerized, activated receptor is rapidly internalized and degraded [17]. Structurally, FLT3 may be divided into 4 distinct domains. Beginning extracellularly and moving inward, these components are (1) the extracellular N terminal, (2) the transmembrane domain, (3) an intracellular, juxtamembrane domain, and (4) an intracellular C-terminal region [18]. Within this C-terminal, there are two tyrosine kinase domains (tyrosine kinase domain 1; TKD1 and tyrosine kinase domain 2; TKD2), interspersed by an inter-kinase domain [18]. Of the 993 amino acids that make up FLT3, just over half constitute the extracellular domain, and the transmembrane domain lies between amino acids 542 and 564 [19]. Amino acids 610–944 comprise the tyrosine kinase domain; interspersed between the transmembrane and tyrosine kinase domain lies the juxtamembrane domain [19]. Finally, the protein terminates in a 50-amino acid C-terminal [19]. 

In humans, FLT3 expression occurs primarily on CD34+ hematopoietic stem cells and both lymphoid and myeloid progenitor populations; it is largely absent from their differentiated progeny, as well as from erythrocyte-lineage progenitors [20,21,22]. Lower-level expression has also been detected in reticuloendothelial and lymphoid tissue (including the spleen and lymph nodes), likely due to the presence of maturing cells of the macrophage and B-cell lineages, respectively [23,24]. Functionally, FLT3 serves as a critical early regulator of hematopoiesis and is required for the proliferation of hematopoietic stem cells [25]. Apart from these roles, and although FLT3 is not expressed in most differentiated cells, it does appear to play a necessary role in regulating dendritic cell development and activation [26,27]. 

Activation of the FLT3 receptor results in increased signaling via multiple signal transduction pathways associated with cell growth, survival, and proliferation, including RAS/RAF/MAPK/ERK, JAK/STAT, and PI3K/AKT [14,28]. In states of health, FLT3 production and activation are tightly controlled, and its activity is negatively regulated via dephosphorylation of the juxtamembrane domain—e.g., the juxtamembrane domain plays an autoregulatory role [13,29]. Mutations that disrupt the function of the juxtamembrane domain, such as occur in FLT3-mutated AML, cause this tight regulation to be lost, and FLT3 becomes constitutively activated [19]. 

## 3. *FLT3* Mutations

### 3.1. Internal Tandem Duplications

**Figure 2:**
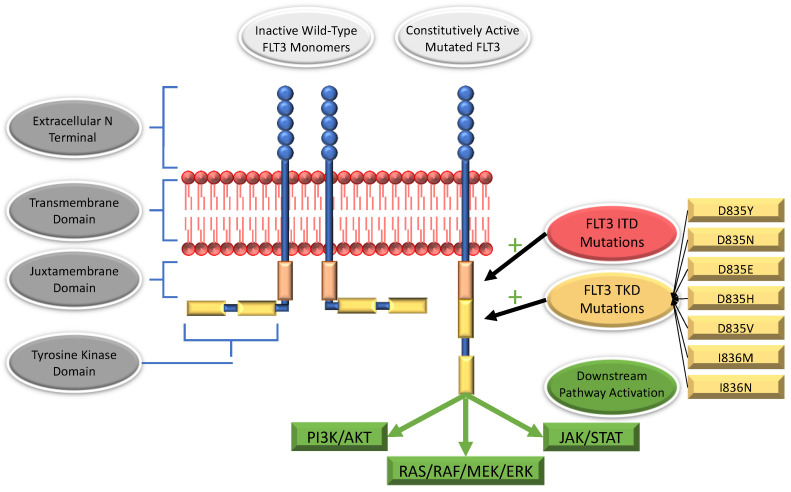
Mutated FLT3 is constitutively active and drives downstream signaling independent of external ligand binding.

*FLT3*-ITD mutations arise as duplications of a variable number of base pairs in exons 14 and 15, which code for FLT3′s juxtamembrane domain [17,30]. These duplications occur in multiples of three, such that the reading frame is preserved (e.g., in-frame) and may range in size from three to several hundred base pairs [17,30]. As a result, additional amino acid sequences are inserted into the juxtamembrane domain, most frequently in the carboxy-terminal region [17]. These mutations compromise the juxtamembrane domain’s usual autoinhibitory function and facilitate ligand-independent dimerization, autophosphorylation, and resultant activation of the receptor (see Figure 2). 

Perhaps counterintuitively, downstream pathway activation differs, however, between “normal” wild-type FLT3 activation (e.g., by FL binding) versus FLT3-ITD-mutated activation. Both result in proliferation and inhibition of myeloid-lineage differentiation. However, compared to wild-type FLT3 activation, FLT3-ITD results in repression of two transcription factors needed for myeloid maturation (PU.1 and C/EBP (CCAAT/enhancer-binding protein)) and inhibition of intracellular phosphatases (including SHP-1 (Src homology region 2 domain-containing phosphatase-1)) [31,32,33]. FLT3-ITD also induces constitutive activity within the WNT signaling pathway via enhanced expression of the WNT ligand’s receptor, FRZ-4 (Frizzled-4) [34]. This activity, and in particular the resultant accumulation of high levels of beta-catenin, occurs in the absence of WNT-ligand when induced by FLT3-ITD. Combined, these alterations therefore result in enhanced anti-apoptotic, self-renewal, and proliferative effects as compared to wild-type FLT3 activation. STAT5 phosphorylation is also discriminant between FLT3 wild-type and ITD-related activation; in the latter case, STAT5 is more highly phosphorylated and more readily acts as a DNA transcription factor, thereby enhancing its growth-promoting and anti-apoptotic effects [35].

A subset of FLT3-ITDs occur within the tyrosine kinase domain; these are distinct from TKD mutations [36,37]. FLT3-ITDs within the tyrosine kinase domain account for up to one-third of all ITDs and, as is seen in juxtamembrane-ITDs, portend a poor prognosis [36,37].

### 3.2. Mutations within the Tyrosine Kinase Domain

Tyrosine kinase domain (TKD) mutations arise due to missense mutations in exon 20, which result in the replacement of a single amino acid residue. At least half of all TKD mutations involve a single codon: aspartic acid 835 (D835); isoleucine 836 (I836) is also relatively commonly implicated [17]. Substitutions of aspartic acid 835 for tyrosine (D835Y) are most common, although asparagine (D835N), glutamate (D835E), histidine (D835H), and valine (D835V) have all been reported [17,38,39]. Isoleucine 836 is most commonly replaced by methionine (I836M) or asparagine (I836N) [17,39]. The implicated amino acids play a role in maintaining monomeric FLT3′s inactive confirmation—their substitution allows constitutive, ligand-independent activation. Interestingly, although FLT3-TKD does mediate increased signaling via the RAS/RAF/MEK/ERK, JAK/STAT, and PI3K/AKT pathways, it does not appear to repress the inhibitory transcription factors PU.1 and C/EBP [40].

Perhaps as a consequence of their resultant lesser effect on pro-survival/anti-apoptotic pathways, the prognostic significance of *FLT3*-TKD mutations is less clearly defined than that of FLT3-ITD mutations, although it appears that patients with FLT3-TKD mutations have superior outcomes to those with *FLT3*-ITD mutations [6,7,8,41,42]. However, the precise effects reported are somewhat variable. In a study examining 676 adult patients with de novo AML, 34 (5%) were identified as FLT3-TKD positive [6]. These patients had superior overall survival (OS) and relapse-free survival (RFS) versus those with FLT3-ITD, and no difference from patients with wild-type FLT3. A second study screened 3082 adult patients, finding 147 (4.8%) with TKD mutations [7]. When analyzed as a whole, FLT3-TKD did not influence either OS or (event-free survival) EFS, both of which were superior when compared to patients with FLT3-ITD. However, the presence of both FLT3-TKD and MLL rearrangements appeared to have a cooperative negative effect on prognosis and worsened outcomes compared to MLL alone. Conversely, FLT3-TKD appeared to have a positive, cooperative effect with NPM1, and improved prognosis. 

### 3.3. Non-ITD, Non-TKD FLT3 Mutations

In addition to the well-described abnormalities discussed above, all mutation classes have been observed within the juxtamembrane domain, including missense, nonsense, deletions, and insertions [43]. On the basis of the existing evidence, it appears that all of the known juxtamembrane domain mutations impair its inhibitory function, e.g., they all increase FLT3 activation/autophosphorylation to varying degrees [43,44]. However, the resultant downstream signaling does not appear to be as robust as that conferred by canonical *FLT3*-ITD mutations [43]. It is important to note, however, that these studies (and the database from which they are drawn [44]) are derived from adult patients with malignancies, and there is, therefore, likely to be a powerful selection bias towards the detection of oncogenic mutations. Moreover, an insufficient number has been detected to allow accurate prognostication of the clinical or biological significance of any one specific mutation.

### 3.4. All ITD Mutations Are not Created Equal

As noted, *FLT3*-ITD mutations are not monolithic, and their presence/absence is not dichotomous. Compared to the relatively homogenous *FLT3*-TKD mutations, in which D835 and I836 mutations account for the vast majority of cases, *FLT3*-ITD mutations are quite varied. Several factors appear important, both biologically and prognostically: the allelic ratio (AR) and the length of the specific mutation present. Additionally, the presence of co-occurring mutations is also significant.

The co-occurrence of both *FLT3*-TKD and -ITD mutations has also been observed in a small proportion of adult patients, generally <1% of those with *FLT3*-mutated AML [6,7,41,45]. Co-occurrence may be more common in children, with observed incidence rates of up to 6% of cases [46]. Although case numbers are insufficient to authoritatively prognosticate, it appears that outcomes are more similar to those seen among patients with isolated *FLT3*-ITD lesions.

#### 3.4.1. Allelic Ratio

In FLT3-ITD AML, the allelic ratio (AR) (or allele level) is the number of ITD-mutant alleles as compared to the number of wild-type alleles present [47]. The AR reflects the number of malignant versus non-malignant cells, as well as the number of mutant alleles within each cell [47]. It is something of an imperfect marker, as it is influenced not only by the actual or “real” biologic AR, but also by the number of blasts present and the presence of any contaminating cells. In most cases, the predominant AML clone is heterozygous for the *FLT3*-ITD mutation, although subclones may be biallelic, hemizygous, possess a different specific mutation, or lack the *FLT3*-ITD mutation altogether; such alterations also affect the AR [48,49]. It is unclear at which specific “threshold” the AR becomes deleterious, but higher ARs (e.g., a greater proportion of mutant-to-wild-type alleles) are correlated with an adverse prognosis [50,51,52,53,54]. Other studies have not shown a marked difference between high and low ARs [51,55], with patients with *FLT3*-ITD mutations having poorer outcomes than those without *FLT3* mutations, irrespective of the AR. The absence of detectable wild-type *FLT3* portends a particularly grim outcome [49,56]. 

One particularly revelatory study examined 630 children with de novo AML enrolled in the Children’s Cancer Group (CCG) studies CCG-2941 and CCG-2961 [28]. Among that cohort, 77 (12%) had *FLT3*-ITD AML, with ARs ranging from 0.01 to 7.5 and a median of 0.53. For children with an AR < 0.43, 4-year PFS was 64%, versus 15% for those with an AR of 0.43–0.64, and 18% with an AR of >0.64 (*p* = 0.014). Moreover, compared to patients who were *FLT3*-WT, children with ITD ARs > 0.4, >0.5, and >0.6 were at an elevated risk for disease progression with hazard ratios of 2.5 (*p* < 0.001), 2.5 (*p* < 0.001), and 2.1 (*p* = 0.001), respectively. Utilizing a threshold of >0.4 as delineating a high ITD AR, patients with an AR greater than 0.4 demonstrated a 4-year PFS of 16% versus 55% among those who were *FLT3*-WT. Patients with low ARs (e.g., 0.4 or less) had similar 4-year PFS to those who were *FLT3*-WT (72% versus 55%, *p* = 0.420). This threshold was subsequently validated based on a re-analysis of a previously reported trial [57]; similar findings were observed, with 3-year overall survival (OS) of patients with an ITD AR of greater than 0.4, 0.4 or lower, and *FLT3*-WT of 20%, 71%, and 63%, respectively (*p* < 0.001) [28]. The subsequent Children’s Oncology Group (COG) phase III randomized clinical trial (AAML0531) therefore utilized an AR of >0.4 to delineate high-risk patients [58]. 

However, more recently updated analyses of these studies have shown that the presence of *FLT3*-ITD mutations, irrespective of the AR and even when between 0.1 and 0.4, portends a poor prognosis [59]. These poor outcomes were effectively unmasked by excluding patients with co-occurring, risk-modifying mutations (specifically CEBPA, NPM1, NUP98-NSD, and WT1); without these patients included in the analysis, EFS from study entry was 25% with an AR of 0.1–0.4, versus 30% for an AR of >0.4; *p* = 0.853. Reflecting this more recent knowledge, the COG’s current phase III clinical trial (AAML1831) includes children with newly diagnosed *FLT3*-mutated AML and defines *FLT3*-ITD as any AR greater than 0.1 (NCT04293562) [60]. Specific sub-analyses will examine outcomes for those with high ARs (e.g., >0.4) versus those with lower ARs, but these patients will not be treated differently. 

#### 3.4.2. Mutation Length

The length of the specific ITD mutations may influence disease outcomes. Depending on the study, the median *FLT3*-ITD size may be 39–61 base pairs; size ranges are quite heterogeneous, ranging from 6 to 210 base pairs in the referenced studies [45,50,61,62]. One study in children identified significantly poorer OS and disease-free survival (DFS) with an ITD length of greater than 48 base pairs [45]; a separate adult study identified medium-length (48–60 base pairs) ITDs as having worse OS and DFS than those with shorter (<48) or longer (>60) mutations [61]. A third study of adults showed no difference in OS or DFS based on ITD length above or below that study’s median length of 61 base pairs but did observe lower remission rates in those with mutation lengths <61 base pairs [50]. Finally, a fourth pediatric study did not identify any association between OS or DFS and ITD mutation length [62].

#### 3.4.3. Co-Occurring Mutations

The mutational landscape of *FLT3*-ITD-mutated AML is heterogeneous, with an average of 13.6 coding mutations (e.g., single nucleotide variants and indels) reported in one large study, which is roughly comparable to AML generally (approximately 10 coding mutations per case) [63,64]. These mutations often, but not always, tend to occur in AML-associated oncogenes, including *nucleophosmin 1, (NPM1), isocitrate dehydrogenase 1 and 2 (IDH 1, IDH 2), Wilms Tumor 1 (WT1), Runt-related transcription factor 1 (RUNX1), Tet methylcytosine dioxygenase 2 (TET2)*, and *DNA (cytosine-5)-methyltransferase 3A (DNMT3A)* among others. Moreover, these mutations tend to persist between initial diagnosis and relapse, hinting at a high degree of stability, particularly *DNMT3A*, *NPM1*, *IDH2*, *RUNX1*, and *TET2* [63,64]. It is, therefore, perhaps unsurprising that the presence of co-occurring mutations may modify the prognosis and outcomes seen in patients with FLT3-mutated AML.

Despite the poor prognosis generally seen among patients with *FLT3*-ITD mutations, in some contexts, its presence may actually prove beneficial, even when the AR is high. The presence of t(6;9)(p23;q34)/*DEK-NUP214* is one such example. This highly deleterious mutation co-occurs with *FLT3*-ITD in up to two-thirds of pediatric cases of AML and is associated with poor outcomes irrespective of *FLT3*-ITD mutation status [65]. Moreover, a range of ARs is seen among patients with t(6;9), although most adult patients have ARs greater than >0.4, e.g., a high AR [66]. In contemporaneous pediatric clinical trials (e.g., COG AAML03P1, AAML0531, and AAML1031 studies), patients with *FLT3*-ITD mutations were considered “high-risk” and underwent allogeneic hematopoietic stem cell transplantation (HSCT) (in studies AAML03P1, AAML0531, and AAML1031) as well as receiving the first-generation FLT3 inhibitor sorafenib (in study AAML1031). Retrospective analysis of children with t(6;9) revealed *superior* outcomes when a co-occurring *FLT3*-ITD mutation was present [66].

Conversely, the effect of co-occurring NPM1 and *FLT3*-ITD mutations appears to be largely predicated on the *FLT3*-ITD AR and to be partially related to age as well. Adult patients with an AR of less than 0.5 have broadly similar outcomes to those with an NPM1 mutation only, e.g., the presence of an NPM1 mutation may have something of a mitigating effect but only when the AR is low [67,68,69,70]. ARs of 0.5 or greater appear to supersede any benefit from the NPM1 mutation [67,68,69,70]. Given the complexity and lack of standardization in determining the *FLT3*-ITD AR, it has been advocated that the co-occurrence of NPM1 and *FLT3*-ITD mutations, even in the presence of a low AR, not be used as an indication to de-intensify therapy or to avoid HSCT when otherwise [8,71]. In contrast, the co-occurrence of *FLT3*-TKD and NPM1 mutations appears to delineate a generally favorable prognosis—in at least two studies of adult patients, those with both lesions appeared to have a superior prognosis as compared to those with either mutation in isolation [7,72]. 

Unlike in adult patients, among children with co-occurring NPM1 and *FLT3*-ITD mutations, the literature suggests that superior outcomes are seen compared to those with *FLT3*-ITD mutations alone, irrespective of the AR [59]. In the cited study, EFS from study entry was 25% for patients with *FLT3*-ITD alone and an AR of 0.1–0.4, and 30% for an AR of >0.4 (*p* = 0.853), but was 70% for those with co-occurring NPM1 or CEBPA plus a *FLT3*-ITD mutation (*p* < 0.001) [59]. These superior outcomes have led to the possibility of (a) omitting HSCT from treatment and (b) utilizing FLT3-inhibitor-based maintenance therapy for this cohort of patients, provided that they achieve end-of-induction remission. This approach is currently under evaluation in the COG’s current phase III clinical trial (AAML1831) (NCT04293562) [60].

As compared to patients with an isolated *FLT3*-ITD mutation, co-occurrence of a *FLT3*-ITD plus a *NUP98-NSD1* and/or *WT1* mutation portends an even worse prognosis [59]. Among a large pediatric cohort of patients treated without FLT3 inhibitors, children with or without *FLT3*-ITD mutations experienced an EFS of 31% versus 48%, respectively, from study entry, versus 17% among those with *FLT3*-ITD mutations plus a *NUP98-NSD1* and/or *WT1* mutation (*p* < 0.001). [59]. These findings were consistent irrespective of the AR, and patients with an AR below 0.4 did not have improved outcomes compared to those with an AR of over 0.4. It, therefore, appears that the *FLT3*-ITD AR loses prognostic significance when paired with these co-occurring mutations, with patients faring poorly irrespective of the specific AR. 

Both pediatric and adult patients with APL demonstrate *FLT3*-ITD at higher rates than in the general AML population (up to 40% of patients with APL versus approximately 25% of all patients with AML) [73]. The presence of *FLT3* mutations is associated with an elevated white blood cell (WBC) count at diagnosis [10], and it was initially believed that the presence of *FLT3*-ITD mutations did not adversely influence outcomes directly among patients with APL [74]. However, more recent analysis has suggested that the presence of an *FLT3*-ITD mutation does indeed portend a poorer prognosis, although to a lesser extent than in AML generally [75]. *FLT3*-TKD mutations also have been associated with poorer outcomes in APL [7]. However, in both cases, it is difficult to ascertain whether these poorer outcomes are directly related to *FLT3* mutational status or with the higher WBC at diagnosis, with which both *FLT3*-ITD and -TKD mutations have been associated [7,75]. The impact is further obfuscated by the fact that the presence of *FLT3*-ITD severely impairs the efficacy of all-trans retinoic acid (ATRA) in ATRA/chemotherapy regimens, but the combination of ATRA/arsenic therapy completely restores therapeutic efficacy [73].

### 3.5. Implications of FLT3 Mutations for AML Risk Categorization

Among adult patients with AML, three primary classification schemata exist. The classification systems used by the National Comprehensive Cancer Network (NCCN) [76] and the European LeukemiaNet (ELN) [77] both divide mutations into favorable risk, intermediate risk, and poor/adverse risk. Based on the NCCN criteria, patients with *FLT3*-ITD AML are classified as being at poor risk. The ELN guidelines likewise stratify patients with *FLT3*-ITD AML into the “adverse risk” grouping but are somewhat more specific: only AML with an *FLT3*-ITD AR of greater than >0.5 is included, and only in the absence of mutant nucleophosmin (NPM1). Co-occurrence of an *NPM1* mutation and *FLT3*-ITD with an AR > 0.5 is considered intermediate risk, as is *FLT3*-ITD with an AR < 0.5 in the absence of *NPM1* mutations. *FLT3*-ITD with an AR < 0.5 and a co-occurring *NPM1* mutation falls into the favorable risk category. Finally, the World Health Organization (WHO)’s classification of myeloid neoplasms and acute leukemia [8] does not specifically stratify AML by risk based on specific mutation but includes *FLT3*-ITD as an alteration with clinical significance.

## 4. Clinical Implementation of FLT3 Inhibitors in Adult Clinical Trials 

The FLT3 inhibitors may broadly be divided into a first generation and a second generation. First-generation FLT3 inhibitors are generally less specific in their inhibition of FLT3 and may have additional off-target effects. The first-generation inhibitors are generally quite broad in their effects, with FLT3 being only one of the many tyrosine kinases inhibited by these agents. The second generation of FLT3 inhibitors are both more selective and more potent in their targeting of FLT3 specifically and have a resultantly improved toxicity profile [8]. Additionally, FLT3 inhibitors may be classified mechanistically as either type 1 tyrosine kinase inhibitors (TKIs; e.g., they inhibit the active and inactive conformation of the target molecule) or as type 2 tyrosine kinase inhibitors (e.g., inhibition of the inactive conformation only; see Table 1) [78,79]. The type 1 FLT3 inhibitors (e.g., crenolanib, gilteritinib, lestaurtinib, midostaurin, MRX-2843, and sunitinib) are active versus both FLT3-ITD and FLT3-TKD, whereas type II inhibitors (e.g., quizartinib, ponatinib, pexidartinib, and sorafenib) are only active versus FLT-ITD. As of writing, two FLT3 inhibitors carry FDA approvals for use in FLT3-mutated AML (midostaurin and gilteritinib). 

### 4.1. First Generation, Type 1 FLT3 Inhibitors

#### 4.1.1. Midostaurin

Midostaurin is a first-generation, type 1 FLT3 inhibitor and was approved by the FDA in April 2017 for use in adult patients with newly diagnosed *FLT3*-mutated AML in combination with cytarabine plus daunorubicin induction and cytarabine consolidation [80]. This approval was based on the results of the RATIFY study (NCT00651261), a phase III randomized clinical trial that enrolled 717 patients with newly-diagnosed *FLT3*-mutated AML [81]. Of these patients, 214 had *FLT3*-ITD mutations with an AR of >0.7 (high), 341 had *FLT3*-ITD mutations with an AR of 0.05–0.7 (low), and 162 had *FLT3*-TKD mutations [81]. Participants were randomized to either standard induction therapy (daunorubicin and cytarabine) or consolidation therapy (high-dose cytarabine), with either midostaurin or placebo, administered on days 8–21 of each cycle. Allogeneic HSCT was carried out at the discretion of individual investigators. Following consolidation therapy, a maintenance phase was administered, in which patients received either midostaurin or placebo for twelve, 28-day cycles.

Adverse events observed in the RATIFY study were relatively similar; the midostaurin arm experienced higher rates of grade 3, 4, or 5 anemia (92.7% vs. 87.8%; *p* = 0.03) and grade 3, 4, or 5 rash (14.1% vs. 7.6%, *p* = 0.008) [81]. Median overall survival was superior in the midostaurin arm (74.4 months versus 25.6 months in the placebo arm; *p* = 0.009), as was 4-year overall survival (51.4% versus 44.3% in the placebo group). When analyzed by mutation type, no significant difference was seen between midostaurin and placebo, however, and no difference was seen in the complete remission (CR) rate (58.9% versus 53.5% in the placebo group; *p* = 0.15). Median EFS was also superior among those receiving midostaurin at 8.2 months versus 3.0 months among those receiving placebo (*p* = 0.002), with patients achieving a benefit regardless of *FLT3* subtype. A subsequent posthoc analysis specifically analyzed patients with the *FLT3*-TKD mutation and confirmed the presence of an EFS benefit at 5 years (45.2% vs. 30.1% on the placebo arm; *p* = 0.044) but did not show a superior 5-year OS (65.9% vs. 58.0%; *p* = 0.244) [82]. 

Notably, the RATIFY trial included the addition of midostaurin maintenance following the completion of induction/consolidation therapy (which also included midostaurin), and as such, it was not possible to directly determine in which phase the addition of midostaurin achieved the greatest benefit. 

The effect of HSCT on patient outcomes among those receiving midostaurin is difficult to assess directly. Of the patients enrolled in the RATIFY trial [81], 101 in the midostaurin arm and 81 in the placebo arm underwent HSCT during 1st complete remission. Neither group reached median overall survival, but results suggested a possible benefit to the midostaurin cohort (69.8 months to “not reached” versus 21.8 months to “not reached” in the placebo group; *p* = 0.07). Additionally, 227 patients received an HSCT following the first CR, with no treatment effect observed (*p* = 0.85). When data were censored at the time of HSCT, a 24.3% lower risk of death was seen in the midostaurin arm, and the 4-year overall survival rate was 63.7% versus 55.7% in the placebo arm. This difference, however, was not statistically significant (*p* = 0.08).

The RADIUS trial sought to more directly establish the use of post-HSCT midostaurin [83]. Following allogeneic HSCT performed in 1st CR, 60 adult patients with *FLT3*-ITD AML were randomized to receive either midostaurin twice daily in 12, 4-week cycles, or midostaurin plus “standard of care (SOC)”. Notably, SOC was non-disease directed, e.g., was comprised of prophylaxis against infection and graft-versus-host disease (GVHD). Of the 30 patients enrolled in each arm, 16 patients in the midostaurin arm and 14 in the control arm completed all 12 cycles. The primary study outcome—RFS—was not significantly different between arms (89% in the midostaurin arm versus 76% in the SOC arm, *p* = 0.27). However, when patients were stratified based on the level of FLT3 inhibition, those with inhibition above the median (70%) had superior survival (*p* = 0.048) and a trend toward reducing relapse (*p* = 0.06). Conversely, patients with FLT3 inhibition below the median (e.g., <70% inhibition) had similar survival and relapse risk to the SOC arm (*p* = 0.9 and *p* = 0.92, respectively). GVHD risk was not increased in the midostaurin arm, although serious adverse events were markedly more common (57% versus 30% in the SOC arm) [83]. 

As of writing, midostaurin is being evaluated in at least 20 AML clinical trials, including in both the relapsed/refractory (R/R) and de-novo setting and in combinations with a number of both novel agents and existing standard chemotherapeutic drugs [111]. Assessment of its use as maintenance therapy is also ongoing.

#### 4.1.2. Lestaurtinib 

Lestaurtinib is a first-generation, type 1 FLT3 inhibitor. It was the first FLT3-directed TKI investigated for use in AML [84], and although it was granted an orphan drug designation status by the FDA in 2006, it has not subsequently been approved for use in this context or any other. The clinical trials AML15 and AML17 investigated lestaurtinib in patients with de novo FLT3 AML [85]. Patients were randomized to receive lestaurtinib versus no additional therapy (AML15) or lestaurtinib versus placebo (AML17) following the completion of each course of chemotherapy for a maximum of 4 cycles. Among the 500 included patients, 74% had *FLT3*-ITD mutation, 23% had *FLT3*-TKD mutations, and 2% had both. No significant differences were identified in 5-year OS or RFS in either study, although patients who achieved greater than 85% FLT3 inhibition (based on assessment via plasma inhibitory assay) did demonstrate significantly superior survival and reduced relapse rates [85]. No active clinical trials assessing its use in AML appear on ClinicalTrials.gov [111].

#### 4.1.3. Sunitinib

Sunitinib is a first-generation, type 1 FLT3 inhibitor. It does not currently carry FDA approval for use in AML, but it is approved for use in gastrointestinal stromal tumors, renal cell carcinomas, and pancreatic neuroendocrine tumors. Sunitinib’s use in *FLT3*-mutated AML has been investigated in a phase I/II clinical trial [86], which paired it with standard 7 + 3 cytarabine/daunorubicin induction and cytarabine consolidation, either as a continuous daily regimen or on days 1–7 of each chemotherapy cycle. Among the 22 included patients, 3 experienced dose-limiting toxicities, and 13 (59%) demonstrated CR/CR with incomplete blood count recovery (CRi; 8/14 with *FLT3*-ITD and 5/8 with *FLT3*-TKD). Of the 5 patients who relapsed on treatment, 4 had lost their initial *FLT3* mutations. No active clinical trials assessing its use in AML appear on ClinicalTrials.gov [111].

### 4.2. First Generation, Type 2 FLT3 Inhibitors

#### 4.2.1. Sorafenib

Sorafenib is a first-generation, type 2 FLT3 inhibitor. At present, it carries FDA approvals for use in metastatic hepatocellular and renal cell carcinomas but not for any AML-related indication. The primary clinical study evaluating its efficacy, SORAML, enrolled 276 patients and randomized them to receive 2 cycles of daunorubicin plus cytarabine induction therapy, followed by three cycles of cytarabine consolidation with either sorafenib or placebo being added to all 5 cycles, and as maintenance for a subsequent duration of 12 months [87]. Notably, patients were not required to have *FLT3*-mutated AML, and of the included participants, 46 (17%) had *FLT3*-ITD mutations, with a median AR of 0.46. 

Among all study participants, SORAML identified superior median EFS (21 months versus 9 months in the placebo arm) to a 3-year EFS rate of 40% versus 22%, *p* = 0.013 [87]. When specifically analyzing patients with *FLT3*-ITD AML, of whom an equal number were treated on each study arm, non-significant improvements were seen in RFS (median 18 months versus 6 months) and OS (not reached versus 19 months). A number of serious adverse events were reported—in particular, grade 3 or greater diarrhea, rash, fever, and cardiac events were all more common in the sorafenib arm. Out of a total of 134 patients receiving the drug, 42 patients (31.3%) withdrew from the sorafenib arm for toxicity-related reasons; dose reductions and drug interruptions were needed in 37 patients (27%) and 50 patients (37%), respectively. 

Long-term follow-up data from SORAML has also recently been released [88]. At 5 years, EFS remained superior among patients treated with sorafenib (41% versus 27% in the placebo arm, *p* = 0.011), as did RFS (53% versus 36% in the placebo arm, *p* = 0.035). In patients with *FLT3*-ITD mutations, 5-year outcomes including EFS, RFS, and OS all failed to achieve statistical significance, but showed clear trends towards improved outcomes: 34.8% versus 8.7% (*p* = 0.084), 42.9% versus 22.7% (*p* = 0.172), and 59.7% versus 39.1% (*p* = 0.178), respectively.

The phase II randomized ALLG AMLM16 clinical trial has analyzed the use of sorafenib in 99 newly diagnosed patients with *FLT3*-ITD AML [89]. Sorafenib was administered during induction (idarubicin plus cytarabine) and consolidation (idarubicin plus etoposide) as well as continued for 12, 28-day cycles following completion of therapy. Although no significant differences were observed between study arms regarding EFS or OS, a trend was seen towards superior outcomes in patients with *FLT3*-ITD ARs greater than 0.7.

Sorafenib’s efficacy as post-HSCT maintenance therapy has also been evaluated in several studies [90,112]. The SORMAIN study was a phase II randomized clinical trial that enrolled 83 adult patients with *FLT3*-ITD AML and assigned them to either 24 months of placebo or sorafenib following the completion of HSCT [90]. A significant survival benefit was observed among patients receiving sorafenib: 24-month OS and RFS were 85% and 90.5% in the sorafenib arm and 53.3% and 66.2% in the placebo arm, respectively. These differences were significant, with *p* = 0.002 and *p* = 0.007, respectively [87]. Compared to the SORAML [87] study, SORMAIN [90] identified similar adverse events. A total of 21 of the 43 patients (48.8%) receiving sorafenib required dose reductions, and 9 patients (22%) discontinued the drug for toxicity-related reasons. Grade 3 or greater diarrhea, skin toxicity, and electrolyte derangements were all seen at higher rates in the treatment arm. One additional phase III randomized clinical trial enrolled 202 patients who underwent HSCT for *FLT3*-ITD AML, 100 of whom received sorafenib [112]. Patients received maintenance with sorafenib or placebo from days 30 to 60 following HSCT. Those receiving sorafenib experienced significantly lower rates of relapse than those receiving placebo; 1-year cumulative relapse rates were 7% and 24.5%, respectively (*p* = 0.001), without significant differences in acute or chronic GVHD, infection risk, or hematologic toxicity. 2-year OS and 2-year leukemia-free survival (LFS) were also significantly improved in the sorafenib versus placebo arm, at 82.1% versus 68.0% (*p* = 0.012) and 78.9% versus 56.6% (*p* < 0.0001), respectively. 

The effect of *FLT3*-ITD mutation heterogeneity on response to FLT3 inhibition has recently been analyzed [53]. Although the cited study sought to analyze FLT3 inhibitors in general, 84% of the included patients (131/156) received sorafenib, and only a small number received other FLT3 inhibitors, midostaurin (9/156) or quizartinib (16/156). No significant difference in OS or RFS was noted based on the number or length of *FLT3*-ITD mutations, nor on the AR, or on the presence of a co-occurring *NPM1* mutation. 

As of writing, sorafenib is being evaluated in at least 15 AML clinical trials across a variety of settings, including in both R/R and de-novo *FLT3*-mutated AML and in combinations with a number of both novel agents and existing standard chemotherapeutic drugs [111].

#### 4.2.2. Pexidartinib

Pexidartinib is a first-generation, type 2 FLT3 inhibitor. Although it does not carry approval for AML, pexidartinib was approved by the FDA in August 2019 for use in adult patients with symptomatic tenosynovial giant cell tumors [80]. Pexidartinib was designed specifically to combat the emergence of resistance to FLT3 inhibition via the acquisition of F691 mutations, especially the F691L mutation [91]. The clinical relevance and development of FLT3 inhibitor resistance of this and other mutations are discussed in a subsequent section of this review. Pexidartinib has been evaluated in a phase I/II clinical trial of 90 patients with R/R AML with *FLT3*-ITD mutations [91]. The drug was safe and relatively well tolerated and displayed a 21% overall response rate with an 11% CR rate; 6 patients were able to subsequently undergo HSCT [91]. No active clinical trials assessing its use in AML appear on ClinicalTrials.gov [111].

#### 4.2.3. Ponatinib

Ponatinib is a first-generation, type 2 FLT3 inhibitor. It carries a relatively broad FDA approval for use in adults with chronic myeloid leukemia (CML) in the chronic phase, accelerated phase, or blast phase that has not responded to prior tyrosine kinase inhibitor therapy, as well as in adults with Philadelphia chromosome-positive acute lymphoblastic leukemia (Ph + ALL) which is resistant or intolerant to prior TKI therapy. This approval partially reflects its wide spectrum of activity, including against ABL (ABL proto-oncogene 1, non-receptor tyrosine kinase), c-KIT, C-SRC, FGFR1 (fibroblast growth factor receptor, LYN, PDGFR (platelet-derived growth factor receptor), and VEGF2R (vascular endothelial growth factor receptor) [18]. 

Ponatinib is able to partially inhibit F691L mutations [92] and was initially proposed as a possible agent to overcome quizartinib resistance via this route. Several early-phase clinical trials have assessed its use in patients with *FLT3*-ITD AML in the R/R setting as monotherapy [93,94] and in combination with azacitidine (the PON-AZA study) [95]. The drug was well-tolerated in the included patients, with a similar safety profile to that seen in CML and response rates approaching 50% in both trials.

Several clinical trials of ponatinib are ongoing in the AML context, including assessments of its use for relapse prevention following stem cell HSCT [113], as part of multiagent therapy for Philadelphia chromosome-positive AML [114], and in children with R/R disease [115]. As of writing, it has not been approved for use in AML, however.

### 4.3. Second Generation, Type 1 FLT3 Inhibitors

#### 4.3.1. Gilteritinib

Gilteritinib is a highly selective, second-generation, type 1 FLT3 inhibitor [96]. It also acts to inhibit the AXL tyrosine kinase receptor, which has been implicated in the development of resistance to FLT3 inhibitors [96,97]. Gilteritinib has shown efficacy in both the *FLT3*-ITD and -TKD contexts [98,99], and was approved by the FDA in November 2018 for use in adult patients with R/R *FLT3*-mutated AML [80]. This approval was based largely on the success of the phase III randomized ADMIRAL trial [99]. The study included 371 patients, of whom 247 were assigned to receive gilteritinib monotherapy and 124 were assigned to receive salvage chemotherapy (mitoxantrone, etoposide, and cytarabine, or fludarabine, cytarabine, granulocyte colony-stimulating factor, and idarubicin, or low-dose cytarabine, or azacitidine). Eligible patients underwent HSCT, followed by post-HSCT gilteritinib maintenance therapy. Patients with *FLT3*-ITD (328 patients; 88%), -TKD (31 patients; 8%), or both (7 patients; 2%) were eligible for inclusions; the median AR was 0.77, with patients above this threshold being considered *FLT3*-ITD high, versus those below who were considered *FLT3*-ITD low. 

Results showed a significant survival benefit among patients treated with gilteritinib—median OS was 9.3 months vs. 5.6 months for patients receiving chemotherapy (*p* < 0.001). One-year survival rate was 37.1% on the gilteritinib arm versus 16.7% in the chemotherapy group. These benefits over chemotherapy were maintained whether chemotherapy was low or high intensity and in patients with either a high or low AR. A higher proportion of patients treated with gilteritinib achieved CR with either full or partial hematologic recovery, at 34.0%, versus 15.3% in the chemotherapy arm (risk difference 18.65; 95% CI, 9.8–27.4%). Response rates and remission duration were similar irrespective of *FLT3* mutations (e.g., TKD or ITD) and, in both cases, were superior to chemotherapy. Subsequently, study results have been updated and continue to show similar overall results, persisting to at least 2 years following completion of therapy [100]. 

The safety profile was quite good—notably, more patients reported serious adverse events on the chemotherapy arm versus the gilteritinib arm. Most grade 3 or greater adverse events among patients receiving gilteritinib were hematological in nature (e.g., febrile neutropenia (45.9%), anemia (40.7%), thrombocytopenia (22.8%)), or involved alanine aminotransferase aspartate aminotransferase. Prolonged QT interval was observed in 4.9% of patients, but only 0.4% (one patient) had a QT greater than 500 msec. Long-term follow-up data did not reveal any additional safety signals [100]. 

Beyond its use in the R/R setting, gilteritinib has also been investigated in a number of trials involving de-novo *FLT3*-mutated AML. In one such phase 1 study, gilteritinib was added to standard 7 + 3 induction and high-dose cytarabine consolidation chemotherapy and continued as maintenance monotherapy [101]. The combination was safe and well-tolerated, with strong evidence of efficacy observed. Preliminary data are available for an ongoing phase III clinical trial comparing gilteritinib versus azacitidine versus gilteritinib plus azacitidine in patients ineligible for intensive chemotherapy [116]. A CR rate of 67% (10/15 patients) has been reported, with accrual ongoing and no new safety signals reported. 

Multiple randomized clinical trials of gilteritinib are ongoing and aim to assess its effectiveness as monotherapy (NCT02752035; phase III) [117], as maintenance therapy with (NCT02997202; phase III) [118] or without (NCT02927262; phase II) [119] preceding HSCT. Additionally, the use of induction/consolidation midostaurin versus gilteritinib is being directly compared [120]. 

#### 4.3.2. Crenolanib

Crenolanib is a second-generation, type 1 FLT3 inhibitor. Although it does not currently carry an FDA designation or approval for AML or any other disease, its development is ongoing. In R/R *FLT3*-mutated AML, preliminary results of an ongoing clinical trial have shown crenolanib monotherapy to have reasonable efficacy, particularly in FLT3-inhibitor-naive patients (39% CR and median survival of 8 months), with a tolerable safety profile [102]. Additionally, in this setting, the combination of crenolanib plus mitoxantrone and cytarabine was shown to be safe in a small cohort of 8 patients, 6 of whom achieved CR [103]. Crenolanib has also been paired with intensive salvage chemotherapy, and overall response rates of 50% were achieved without major adverse events [104]. In patients with de novo *FLT3*-mutated AML, a phase II trial of crenolanib combined with standard induction/consolidation therapy involving 29 patients achieved an 83% (24/29) CR rate; 2 patients later relapsed [105]. Finally, post-HSCT maintenance therapy appears to be both safe and well-tolerated, with further phase III studies planned [121].

At present, crenolanib is being evaluated in a phase III randomized clinical trial comparing it to midostaurin for use in newly diagnosed patients with *FLT3*-mutated AML [122], as well as in a phase III randomized clinical trial comparing salvage chemotherapy with or without crenolanib for patients with R/R *FLT3*-mutated AML [123].

#### 4.3.3. MRX-2843

The development of MRX-2843 followed that of UNC1666—both compounds are type 1 inhibitors of MERTK (MER proto-oncogene, tyrosine kinase) and FLT3. Briefly, MERTK is a receptor tyrosine kinase that is overexpressed in the vast majority of AML samples, and inhibition of this receptor has been shown to reduce pro-survival/anti-apoptotic signaling [106]. UNC1666 showed promising in vitro activity but had poor bioavailability, and clinical development has not proceeded further [107]. MRX-2843 was therefore developed to improve upon this. Both in vitro and murine models have shown it to effectively abrogate FLT3 signaling, and it is also capable of inhibiting the quizartinib-resistance-inducing mutations D835 and F691 [108]. On the basis of these results, MRX-2843 is currently being evaluated in a phase I study of patients with R/R AML, of whom a subset will have *FLT3*-ITD mutations [124]. One additional phase I/II study is also occurring in R/R AML, with patients enrolled in the phase II portion of that trial being required to have either *FLT3*-ITD or TKD mutations [125].

### 4.4. Second Generation, Type 2 FLT3 Inhibitors

#### Quizartinib

Quizartinib is a second-generation, type 2 FLT3 inhibitor. In the AML context, it is currently approved for use in Japan but does not presently carry FDA approval in the US; although it was designated a breakthrough therapy in August 2018, the FDA declined to approve its use in *FLT3*-mutated AML in June 2019 [80,126]. This was based largely on concerns regarding cardiotoxicity, as well as concerns regarding the magnitude of benefit demonstrated in the QuANTUM-R clinical trial [109]. This phase III study randomized patients with R/R *FLT3*-ITD AML to single-agent quizartinib or to salvage chemotherapy using any of the following: low-dose cytarabine, or mitoxantrone/etoposide/cytarabine, or fludarabine/cytarabine/idarubicin granulocyte colony-stimulating factor. In the 367 enrolled patients, OS was superior for quizartinib versus for chemotherapy (*p* = 0.02), but the duration was relatively short, with median overall survival of 6.2 months versus 4.7 months in the chemotherapy arm. Intriguingly, 32% of the patients on the quizartinib arm were able to proceed to HSCT, versus 11% in the salvage chemotherapy group. 

One possible explanation for the relatively disappointing results of the QuANTUM-R study [109] is the use of quizartinib monotherapy instead of as an addition to the existing standard of care. The results of the current phase 3 randomized clinical trial “QuANTUM First” (NCT02668653) [127] will therefore be intriguing. The trial is assessing quizartinib’s use in conjunction with standard of care chemotherapy, and as continuation therapy, in patients with newly diagnosed FLT3-ITD AML. 

The cardiotoxicity of quizartinib has been of concern and factored into the FDA’s decision not to approve it. Prolongation of the QT interval, in particular, has been reported, occurring in up to 17% [110]–22% [109] of patients; however, the incidence of grade 3 or greater QT prolongation appears to be under 5% [109,110]. Other than this notable toxicity, however, quizartinib appears to be safe and well-tolerated. 

## 5. FLT3 Inhibition in Pediatric FLT3-Mutated AML

Pediatric patients with AML generally have a superior prognosis to adult patients with equivalent mutations, and this is true of *FLT3* mutations. Compared to children with *FLT3*-WT, however, *FLT3*-ITD carries a poorer prognosis: prior to the advent of FLT3 inhibitors, progression-free survival (PFS) was approximately 33% at 4 years, with relapse rates of up to 35% [28,128]. *FLT3*-ITD is therefore classified as a high-risk form of AML by cooperative groups such as the Children’s Oncology Group (COG), and consolidative allogeneic HSCT is recommended [128]. The integration of FLT3 inhibition into the modern treatment paradigm has therefore been a major advance, although many difficulties remain in securing access to these agents outside of specific clinical trials [129].

The COG phase III clinical trial AAML1031 included sorafenib, a first-generation FLT3 inhibitor, in all chemotherapeutic courses and as maintenance therapy for one year following HSCT. Patients were considered to be *FLT3*-mutated if they carried an *FLT3*-ITD mutation with an AR of 0.4 or greater. Significant improvements in outcome were seen, including a 3-year EFS of 58% (versus 34% in a comparable historical cohort; *p* = 0.007) and a reduction in 3-year risk of relapse from 52.2% in the historical control arm to 18.2% *p* = 0.006) [130,131]. However, being relatively non-specific, sorafenib was poorly tolerated, and only 25% of children with *FLT3*-ITD AML received maintenance sorafenib according to the study protocol; frequent dose modifications, dose holds, and drug discontinuation occurred [130,131]. Further, significant cardiotoxicity resulted in the study being temporarily paused [52,130,131]. 

Midostaurin, another first-generation FLT3 inhibitor, and quizartinib, a second-generation FLT3 inhibitor, have also been investigated in the pediatric setting. Although data are currently limited to a single phase I/II trial of midostaurin [132] and a single phase I trial of quizartinib [133], both of which occurred in the R/R setting, initial results appear promising, and further clinical trials are underway [134,135]. Crenolanib has also been trialed in a small number of pediatric patients in combination with sorafenib [136]. The combination was well tolerated in this pilot study, which included 9 children with R/R *FLT3*-ITD AML and achieved complete remissions in 3 patients. No ongoing pediatric studies appear on ClinicalTrials.gov [111]. 

Gilteritinib, a second-generation FLT3 inhibitor, is currently undergoing evaluation in children with *FLT3*-mutated AML as well, but definitive data are not yet available. The COG’s phase III AAML1831 clinical trial includes children with newly diagnosed *FLT3*-mutated AML (including both *FLT3*-ITD with an AR of >0.1 and *FLT3*-TKD) (NCT04293562) [60]. Gilteritinib’s efficacy is also being assessed in the context of pediatric R/R *FLT3*-ITD AML (NCT04240002) [137].

## 6. CD33+ Targeting, Consolidative HSCT, and Integration of FLT3 Inhibition

*FLT3*-ITD mutations are associated with increased expression of CD33 [138]. Briefly, CD33 is a marker of myeloid differentiation present on the cell surface of malignant blasts in most cases of AML. It is also a targetable antigen: gemtuzumab ozogamicin (GO) is a drug-antibody conjugate comprised of the DNA-binding cytotoxin calicheamicin and a humanized IgG4, anti-CD33 monoclonal antibody [58]. Its integration into AML therapy has resulted in improved outcomes among patients with newly diagnosed AML, where it has been paired with standard chemotherapy [58]. CD33 expression and response to GO appear to be linked, such that patients with high levels of CD33 experience a pronounced increase in EFS and a reduction in relapse rate; these benefits are seen across AML risk groups [139]. When combined with consolidative HSCT, patients who had *FLT3*-ITD AML and who received GO appear to have a superior prognosis compared to other high-risk patients without this mutation, even in the absence of FLT3 inhibitor therapy [58]. Moreover, patients with *FLT3*-ITD AML who receive GO have superior outcomes as compared to patients who do not receive it; this difference is further increased when paired with a consolidative HSCT, such that patients with newly diagnosed *FLT3*-ITD AML who receive both GO and an HSCT have the best outcomes [140].

The combination of FLT3 inhibition (using gilteritinib), HSCT, and GO is currently being tested in the COG’s phase III AAML1831 clinical trial [60]. Preliminary results are also available for a recent phase I study of midostaurin plus GO in newly diagnosed *FLT3*-mutated AML. Although 5/11 patients (45%) experienced at least one serious adverse event, 10/11 (91%) of patients achieved CR [141].

## 7. Resistance to FLT3 Inhibition—And How to Overcome It 

Despite their undeniable promise in treating patients with *FLT3*-mutated AML, the deployment of FLT3 inhibitors has been met with several challenges. Perhaps the most important of these is the emergence of resistance to FLT3 inhibition. This may occur via any of a number of mechanisms, but as the understanding of these mechanisms continues to expand, novel methods of combatting FLT3 inhibitor resistance are also emerging. The following section has been divided into mechanisms that are intrinsic to AML cells and to those which are emergent, e.g., those which are selected for and may develop during therapy. This is, however, something of a false dichotomy as it belies the complex interplay between the two routes to resistance. 

### 7.1. Intrinsic Mechanisms of Resistance

Although FLT3 inhibitors target *FLT3*-mutated AML, their disruption of signaling via wild-type FLT3 is relatively poor by comparison. Physiologic activation via FLT3 receptor—FLT3 ligand pairing allows downstream pathways to continue to function [142]. Similarly, even when peripheral circulating blasts are no longer detectable, blasts sequestered within the bone marrow are minimally affected, if at all. It appears that this protection is mediated at least partially via the FLT3 ligand, the presence of which has been shown to increase the inhibitory concentration necessary to disrupt FLT3 signaling by at least 50% [143]. *FLT3*-mutated blasts retain the ability to respond to signaling via the FLT3 ligand, and this signaling impedes the effectiveness of FLT3 inhibitors [144].

Pharmacogenomics may also play a role in both resistance and toxicity. As has been noted, a key aspect of FLT3 inhibitor efficacy is the degree to which FLT3 signaling is disrupted, which is itself reflective of drug concentration in vivo. However, FLT3 inhibitors are metabolized by hepatic cytochrome P450 A4 (CYP3A4) [145]. This has direct clinical relevance—drugs that inhibit CYP3A are in widespread use, most notably in this population via the azole class of antifungals [146,147]. Moreover, CYP3A4 polymorphisms which enable rapid or poor metabolism may either diminish efficacy or increase toxicity, respectively. Beyond hepatic expression, however, CYP3A4 has been identified in the bone marrow stroma, where it impairs the activity of FLT3 inhibition in a potentially meaningful fashion [148]. The authors of the cited study showed, intriguingly, that pairing FLT3 inhibitors with CYP3A4 inhibitors (clarithromycin, in this case) significantly abrogates this mechanism of resistance and may be a promising strategy [148]. 

The bone marrow stromal niche may therefore play an outsized and surprising role in response to FLT3 inhibitor therapy. Beyond FLT3 ligand expression [143] and CYP3A4 expression [148], it appears that—unlike peripheral circulating blasts—bone marrow blasts experience cell-cycle arrest rather than apoptosis in response to FLT3 inhibition [143]. Signaling via the RAS/RAF/MEK/ERK pathway appears critically important in the maintenance of these *FLT3*-mutated blasts [143]. However, the addition of pathway-specific inhibitors (such as the MEK inhibitor trametinib) may therefore provide a therapeutic benefit, as has been suggested by in vitro data [143]. This observation also parallels and partially explains the earlier observation that activating RAS mutations facilitated resistance to FLT3 inhibition [149].

### 7.2. Emergent Mechanisms of Resistance

Perhaps the most intuitively understood mechanism of resistance is the emergence of mutations that diminish the function of FLT3 inhibitors; this is directly analogous to the emergence of antibiotic resistance among bacterial species. The first such case was reported in conjunction with midostaurin therapy; in that case, the emergence of a single amino acid substitution within the tyrosine kinase domain (N676K) was sufficient to facilitate resistance in a patient with *FLT3*-ITD AML [150]. Amino acid substitutions at F691 and D835 appear to be more common than at N676 [79,151]. Even in patients with *FLT3*-ITD AML, the presence of these point mutations—occurring outside the juxtamembrane domain—disrupt inhibitor binding to their target sites. It is unclear whether the emergence of leukemic clones bearing these mutations reflects de novo acquisition, selection pressure on a previously undetected minor clone, or the presence of a low-level co-occurring TKD mutation within the predominant *FLT3*-ITD clone. It, therefore, seems possible that the heterogeneity in patient response to FLT3 inhibition may be partially reflective of the heterogeneity within the molecular landscape of *FLT3* mutations.

Beyond mutations that disrupt target-site interactions, it appears that simple exposure to FLT3 inhibitors may be capable of rapidly inducing resistance. FLT3 inhibitors have been shown experimentally to induce increased expression of FLT3 in as little as 4–8 h [96,152]. Intriguingly, the novel agent fimepinostat (CUDC-907), a dual PI3K and histone deacetylase (HDAC) inhibitor, has been shown to be capable of downregulating FLT3 [153]. Experimentally, the combination of fimepinostat plus gilteritinib exerts synergistic antileukemic effects partially mediated via the abrogation of FLT3-inhibitor-induced FLT3-upregulation [152]. Although not yet deployed clinically, this combination raises the possibility of directly targeting FLT3 resistance by blocking the mechanisms by which it may arise. 

Apart from mutations in the targeted receptor and altered expression of that receptor, leukemic cells are also able to escape FLT3 inhibition by effectively bypassing reliance upon this receptor. Activation of the FLT3 receptor results in increased signaling via multiple signal transduction pathways associated with cell growth, survival, and proliferation, including RAS/RAF/MAPK/ERK, JAK/STAT, and PI3K/AKT [14,28]. However, these pathways do not rely solely upon FLT3 for their activation and are activated by a number of cell-surface receptors. One such receptor is AXL, a member of the TAM receptor tyrosine kinase family (somewhat recursively named; TAM standing for “Tyro3, Axl and MerTk”). AXL is activated by growth arrest-specific gene 6 (GAS6) and, like FLT3, promotes cell survival and proliferation via multiple downstream pathways, many of which overlap or are the same as those activated by FLT3 [154]. AXL has been implicated in FLT3 inhibitor resistance: signaling has been shown to be upregulated in the presence of quizartinib and midostaurin [154]. Conversely, AXL blockade synergistically enhances the antileukemic activity of FLT3 inhibitors [152,154]. This may partially explain the discordant study results observed between gilteritinib versus quizartinib. The ADMIRAL trial [99] and the QuANTUM-R trial [109] were similar insofar as their comparison of FLT3 inhibitor monotherapy to salvage chemotherapy in the R/R FLT3-AML setting. However, gilteritinib inhibits both AXL and FLT3 [152], and this difference may partially account for the superior outcomes seen with that agent. Further downstream, fimepinostat, discussed previously, has also been shown to exert synergistic antileukemic effects when combined with FLT3 inhibition via blockade of both the JAK/STAT and RAS/RAF/MEK/ERK pathways [152]. These results suggest the utility of multi-pathway inhibition in overcoming FLT3 inhibitor resistance. 

FLT3′s anti-apoptotic effects are partially mediated via its influence on the B-cell lymphoma 2 (BCL-2) family of proteins. Briefly, this family, named for the eponymous BCL-2 protein, is critical to the regulation of the mitochondrial membrane potential (MMP) [155]. Disruption or loss of MMP results in apoptosis via the release of proapoptotic proteins previously sequestered within the mitochondrial membrane, which, upon their release, initiate a cascade resulting in the eventual destruction of double-stranded DNA [155]. The members of the BCL-2 family maintain a tightly regulated balance between pro-and anti-apoptotic proteins—disruption of this balance may tilt a cell either towards apoptosis or provide it with a potent means of resisting pro-apoptotic signaling [155]. 

BCL-2 inhibition is an emerging therapeutic area, and effective counters to this resistance mechanism exist. Venetoclax is a BH3-mimetic and selective inhibitor of BCL-2 [156]. It was granted accelerated approval by the FDA in November 2018 for use as part of first-line treatment of de novo AML adults aged 75 years or older or those unable to tolerate standard, intensive chemotherapy [156]. Venetoclax, via its mechanisms as a BCL-2 inhibitor and BH3 mimetic, effectively tilts the intracellular balance irrevocably towards apoptosis [155]. This agent, when combined with FLT3 inhibition, shows notable antileukemic synergy in vitro via disruption of BCL-2 signaling, somewhat restoring, in effect, the pro/anti-apoptotic balance disrupted by *FLT3* mutations. Somewhat elegantly, ERK induction by venetoclax may be one mechanism of resistance to that drug, but its upregulation is entirely abrogated via the addition of FLT3 inhibitors [157].

Venetoclax also synergizes well with a wide range of other antileukemic agents currently in development for AML, such as vosaroxin (a second-generation topoisomerase II inhibitor and DNA intercalating agent) [158], fimepinostat (a dual inhibitor of phosphatidylinositol 3-kinases (PI3K) and histone deacetylases (HDACs)) [159], selinexor (an exportin 1 (XPO1) inhibitor) [160], and SLC-391 (a highly selective inhibitor of tyrosine-protein kinase receptor UFO; AXL) [161]. This raises an intriguing possibility: the addition of venetoclax could synergistically enhance the efficacy of multiple therapeutic agents *simultaneously*. Although this review has focused on *FLT3*-mutated AML, venetoclax appears to be agnostic insofar as its effects on any particular mutation; patients with *NPM1*, *DNMT3A*, *FLT3*-ITD, and *SRSF2* mutations have all demonstrated excellent responses. As discussed above, resistance to FLT3 inhibition may develop via the bypassing of any one point of disruption. Simultaneous inhibition of multiple pathways is a means by which resistance could therefore be abrogated. Having been recently integrated into frontline AML trials [162,163,164], the combination of venetoclax plus FLT3 inhibition is currently undergoing evaluation in multiple clinical trials for the treatment of *FLT3*-mutated AML [165,166,167].

## 8. Conclusions and Future Directions

FLT3 inhibitors have proliferated rapidly, as has the advent of clinical trials assessing their use in *FLT3*-mutated AML. Although this disease remains a particularly deadly malignancy, FLT3 inhibitors are beginning to change the treatment paradigm and are providing clear improvements in patient outcomes. However, many challenges remain, and their full integration into the *FLT3*-mutated AML treatment paradigm will depend on overcoming these obstacles. 

The development of resistance, as discussed above, will continue to pose a threat and require ongoing innovation. FLT3 inhibitor monotherapy appears to be particularly vulnerable, and combinatorial approaches are likely to be necessary. A related problem is the heterogeneity of *FLT3* mutations: FLT3 inhibitors are not monolithic, and thoughtful selection of the optimal agent to target a patient’s mutation may become necessary to optimize outcomes. Recent literature has also highlighted the importance of co-occurring mutations and underscored the relatively lessened importance of the *FLT3*-ITD AR in this context; certainly, the AR is superseded by the presence of specific co-occurring mutations. 

At present, it is not possible to directly contrast the FLT3 inhibitors, as head-to-head trials are lacking. However, a number of existent clinical trials are seeking to do precisely that. It is to be hoped that clear efficacy signals will emerge, so as to guide the selection of the most effective agents. A growing number of FLT3 inhibitors are available, with additional agents in development, and as the landscape becomes more crowded, it will become increasingly necessary for clinicians to decide among therapeutic options. Similarly, although the side-effect profiles of the FLT3 inhibitors are relatively similar, they are not identical—a situation typified by the apparent heightened arrhythmogenic potential of quizartinib as compared to other agents of the class. Although much work remains to be done, with the advent of FLT3 inhibitors, it is to be hoped that these lesions may soon lose their prognostic significance. This is especially true as these agents become more selective, are moved to frontline therapy, and are integrated with other therapies that turn the presence of *FLT3* mutations into an advantage, such as has recently been seen with GO. 

The ever-growing understanding of the molecular drivers of malignancy has revolutionized cancer therapy. As these lesions become targetable, patient outcomes have markedly improved. Although much work remains, it is to be hoped that *FLT3*-mutated AML will soon join CML and APL as diseases that, while once near-uniform in their lethality, are now readily treatable via the use of precisely targeted therapies. 

## Figures and Tables

**Table 1:** FDA-approved and in-development FLT3 inhibitors, displayed by generation (first or second) and tyrosine kinase inhibitor type (type 1 or type 2).

	Type 1 FLT3 Inhibitors (Inhibition of Both Active and Inactive FLT3 Confirmation)	Type 2 FLT3 Inhibitors (Inhibition of Inactive Conformation Only)
**First Generation** **FLT3 Inhibitors**	* Midostaurin [80,81,82,83] Lestaurtinib [84,85] Sunitinib [86]	Sorafenib [87,88,89,90] Pexidartinib [91] Ponatinib [92,93,94,95]
**Second Generation** **FLT3 Inhibitors**	** Gilteritinib [96,97,98,99,100,101] Crenolanib [67,102,103,104,105] MRX-2843 [106,107,108]	Quizartinib [109,110]

* Midostaurin is FDA-approved for adult patients with newly diagnosed *FLT3*-mutated AML, in combination with cytarabine plus daunorubicin induction and cytarabine consolidation. ** Gilteritinib is FDA-approved for adult patients with relapsed/refractory *FLT3*-mutated AML.

## Data Availability

All available data are provided in the manuscript.
